# Impact of Elevated CO_2_ and Reducing the Source-Sink Ratio by Partial Defoliation on Rice Grain Quality – A 3-Year Free-Air CO_2_ Enrichment Study

**DOI:** 10.3389/fpls.2021.788104

**Published:** 2021-12-23

**Authors:** Bo Gao, Shaowu Hu, Liquan Jing, Yunxia Wang, Jianguo Zhu, Kai Wang, Hongyang Li, Xingxing Sun, Yulong Wang, Lianxin Yang

**Affiliations:** ^1^Key Laboratory of Crop Genetics and Physiology of Jiangsu Province, Co-Innovation Center for Modern Production Technology of Grain Crops of Jiangsu Province, Yangzhou University, Yangzhou, China; ^2^Jiangsu Coastal Area Institute of Agricultural Sciences, Yancheng, China; ^3^College of Environmental Science and Engineering, Yangzhou University, Yangzhou, China; ^4^State Key Laboratory of Soil and Sustainable Agriculture, Institute of Soil Science, Chinese Academy of Sciences, Nanjing, China

**Keywords:** climate change, *Oryza sativa*, quality, free-air CO_2_ enrichment, source and sink

## Abstract

Evaluating the impact of increasing CO_2_ on rice quality is becoming a global concern. However, whether adjusting the source-sink ratio will affect the response of rice grain quality to elevated CO_2_ concentrations remains unknown. In 2016–2018, we conducted a free-air CO_2_ enrichment experiment using a popular *japonica* cultivar grown at ambient and elevated CO_2_ levels (eCO_2_, increased by 200 ppm), reducing the source-sink ratio *via* cutting leaves (LC) at the heading stage, to investigate the effects of eCO_2_ and LC and their interactions on rice processing, appearance, nutrition, and eating quality. Averaged across 3 years, eCO_2_ significantly decreased brown rice percentage (−0.5%), milled rice percentage (−2.1%), and head rice percentage (−4.2%) but increased chalky grain percentage (+ 22.3%) and chalkiness degree (+ 26.3%). Markedly, eCO_2_ increased peak viscosity (+ 2.9%) and minimum viscosity (+ 3.8%) but decreased setback (−96.1%) of powder rice and increased the appearance (+ 4.5%), stickiness (+ 3.5%) and balance degree (+ 4.8%) of cooked rice, while decreasing the hardness (−6.7%), resulting in better palatability (+ 4.0%). Further, eCO_2_ significantly decreased the concentrations of protein, Ca, S, and Cu by 5.3, 4.7, 2.2, and 9.6%, respectively, but increased K concentration by 3.9%. Responses of nutritional quality in different grain positions (brown and milled rice) to eCO_2_ showed the same trend. Compared with control treatment, LC significantly increased chalky grain percentage, chalkiness degree, protein concentration, mineral element levels (except for B and Mn), and phytic acid concentration. Our results indicate that eCO_2_ reduced rice processing suitability, appearance, and nutritional quality but improved the eating quality. Rice quality varied significantly among years; however, few CO_2_ by year, CO_2_ by LC, or CO_2_ by grain position interactions were detected, indicating that the effects of eCO_2_ on rice quality varied little with the growing seasons, the decrease in the source-sink ratios or the different grain positions.

## Introduction

Against the background of a changing climate, concentrations of atmospheric carbon dioxide (CO_2_) are increasing and predicted to exceed 900 ppm at the end of this century, with a current value of 410 ppm (Intergovernmental Panel on Climate Change (IPCC), 2021; National Oceanic and Atmospheric Administration (NOAA), 2021). Rice (*Oryza sativa*) is one of the most important food crops globally, and more than half of the global population consumes rice as a staple (Fan et al., 2016). This makes it necessary to investigate the impacts of increasing CO_2_ concentrations on rice quality.

Rice quality can be measured in terms of processing suitability, appearance, eating quality, and nutritional quality. Most studies on changes in rice quality under elevated CO_2_ (eCO_2_) levels have only involved a few of these indicators, such as appearance (Usui et al., 2014) or nutritional quality (Ujiie et al., 2019), with few studies having investigated all the above-mentioned characteristics (Yang et al., 2007). Regarding the response of rice quality to eCO_2_, Chaturvedi et al. (2017) found that eCO_2_ significantly increased the chalkiness of rice, whereas Xu et al. (2008) reported that the chalkiness of a *japonica* “Asominori” and an *indica* “IR24” cross showed a decreasing trend under eCO_2_. The amylose concentration is often used as one of the indicators to evaluate the eating quality of rice (Cameron and Wang, 2005), and in previous studies, the response of the amylose concentration to eCO_2_ either increased (Seneweera et al., 1996; Seneweera and Conroy, 1997), decreased (Yang et al., 2007; Goufo et al., 2014) or remained unchanged (Terao et al., 2005; Hu et al., 2019). In some studies, the variation in rapid visco analyzer (RVA) parameters (such as peak viscosity or setback) under eCO_2_ indicated an improvement of rice eating quality (Allahgholipour et al., 2006; Yang et al., 2007). However, these indicators were measured using rice flour and not rice grains, which are the usually consumed form of rice, and more meaningful results may be obtained by determining the eating value of cooked rice. Terao et al. (2005) showed that the eating value and stickiness of rice increased under eCO_2_, but these results were not statistically significant. In a few studies on rice palatability, Jing et al. (2016a; 2021a; 2021b) reported that eCO_2_ had the tendency to improve the palatability of rice.

Nutritional quality is an important rice index. In previous studies, eCO_2_ resulted in a consistent decrease in protein concentration (Zhang et al., 2013; Zhu et al., 2018; Ainsworth and Long, 2021), thus causing a risk of “hidden hunger” for people who consume rice (Loladze, 2014). However, previous studies have found differences in the effects of eCO_2_ on the mineral concentrations in rice. For example, Myers et al. (2014) found that eCO_2_ could reduce the concentrations of zinc (Zn), iron (Fe), copper (Cu), manganese (Mn), sulfur (S), and other elements by 3–11%, whereas according to Lieffering et al. (2004), an increase in atmospheric CO_2_ concentration of 200 ppm only had a significant negative impact on the nitrogen (N) concentration of rice. More importantly, most current studies on the nutritional quality of rice under eCO_2_ used brown rice as their research object (Lieffering et al., 2004; Zhu et al., 2018); however, when rice is the main nutritional source, it is generally milled. According to Ujiie et al. (2019), the decrease in N, S, Mn, and Zn concentrations in milled rice under eCO_2_ is significantly greater than that in brown rice. Phytic acid is an antinutrient widely present in seeds and fruits and can reduce the effective absorption of Zn and other elements (Miller et al., 2007; Schlemmer et al., 2009); the effect of eCO_2_ on phytic acid concentrations in rice is still unclear (Myers et al., 2014).

The source-sink relationship plays an important role in rice growth (Dingkuhn et al., 2020). Generally, researchers select rice varieties (or strains) with different source-sink types or change the source-sink relationship (ratio) of the same variety by artificial adjustment to understand its relative contribution to crop growth changes. The source strength can usually be changed by cutting off leaves and increasing CO_2_ concentration, whereas the sink strength can be adjusted by removing spikelets or decreasing temperature (Shimono et al., 2008; Jing et al., 2016b). For example, Yuan et al. (2005) and Tao et al. (2006) found that cutting off leaves of rice plants significantly increased chalkiness; whereas Jing et al. (2016b) reported that removing spikelets significantly reduced chalkiness; however, the response trends to increasing CO_2_ concentrations followed the opposite patterns, and there was no interaction between spikelet removal and CO_2_ level. A recent meta-analysis (Ainsworth and Long, 2021) showed that the fertilizer effect of eCO_2_ on rice yield was positively correlated with the storage capacity of the tested varieties. For example, rice cultivars with higher spikelet density showed greater responses of yield to eCO_2_ than those with lower density (Shimono et al., 2009; Hu et al., 2021). Similarly, Nakano et al. (2017) also observed that the yield increase of rice strains with large panicles was significantly greater under eCO_2_ compared with that of the parent plants. Previous studies have found that the fertilizer effects of eCO_2_ on rice growth can be changed by manually adjusting the source-sink ratio *via* cutting leaves and/or removing spikelets. For example, Fabre et al. (2019) found that compared with naturally grown rice, increasing the source-sink ratio of rice by removing spikelets could inhibit the photosynthetic capacity of leaves under eCO_2_. In our previous field study, we showed that reducing the source-sink ratio *via* cutting off leaves at the heading stage could enhance the response of the net photosynthetic rate of the remaining leaves to eCO_2_ at the filling stage and increase the seed filling ability, with significant increases in yield under eCO_2_. In contrast, removing spikelets increased the source-sink ratio, showing the opposite trend (Lai et al., 2016; Gao et al., 2021). However, so far, the effects of eCO_2_ on rice quality under different source and sink treatments are still largely unclear. As a carbon source for photosynthesis, CO_2_ at increasing levels increases the “source,” whereas the leaf source could be artificially reduced by leaf cutting. However, it is unclear whether rice quality is improved by leaf cutting, similar to rice yield, under eCO_2_ environments. Also, the differences in quality response to eCO_2_ between milled and brown rice and changes in these responses with different growing seasons remain unknown.

Studies conducted in air chambers are not suitable to investigate the interaction between source and sink at the crop population level because of their disturbed microclimate and limited space (McLeod and Long, 1999). To avoid these issues, free-air CO_2_ enrichment (FACE) systems with standard crop management techniques, free air flow, large test areas and field environmental conditions can provide an opportunity to study the relationship between the source and sink in rice (Long et al., 2006; Yoshinaga et al., 2020). For this study, we cultivated the super *japonica* cultivar “Wuyunjing 27” in a large paddy FACE site located in Yangzhou, China, from 2016 to 2018. The source-sink ratio was reduced *via* cutting off leaves at the heading stage to construct crops with different source-sink levels and to investigate the effects of eCO_2_ and leaf cutting on rice quality. Our results provide a scientific basis for an improved understanding and predicting rice quality changes under elevated CO_2_ concentrations.

## Materials and Methods

### Experimental Site and Free-Air CO_2_ Enrichment System

This study was conducted at the FACE system in Yangzhou (119°42′0″E, 32°35′5″N), Jiangsu Province, China. The basic soil properties are as follows: soil organic carbon 24.8 g⋅kg^–1^, total nitrogen 1.13 g⋅kg^–1^, total phosphorus 0.54 g⋅kg^–1^, total potassium 9.7 g⋅kg^–1^, available nitrogen 122.4 mg⋅kg^–1^, available phosphorus 15.1 mg⋅kg^–1^, available potassium 56.5 mg⋅kg^–1^, pH 6.9. Details of the FACE platform can be found elsewhere (Yang et al., 2009; Zhu et al., 2018). In brief, the FACE system contains six plots located in different paddies with similar soil and agronomic histories. Three plots were randomly allocated for the elevated CO_2_ treatments and three for the ambient treatments. Each FACE plot was about 80 m^2^, and the distance between the center of the FACE and ambient plots was 90 m to avoid CO_2_ contamination. Pure CO_2_ gas was emitted into the center through pipelines around the FACE plots, and the CO_2_ concentration of the platform was monitored and controlled by a computer network. According to the CO_2_ concentration in the atmosphere, wind direction, wind speed, and CO_2_ concentration at the crop canopy height, the release speed and direction of CO_2_ gas were automatically adjusted to maintain a concentration 200 ppm higher in the main growth period of rice in the FACE plots compared with the ambient condition. The CO_2_ fumigation began after seedling transplanting and continued until plant maturity; the treatment period was from sunrise to sunset. The mean daily average temperatures from June 1 to October 31 were 25.0, 24.9, and 25.5°C in 2016, 2017, and 2018, respectively (Supplementary Figure 1). The average monthly precipitation levels were 274.9, 149.1, and 120.1 mm and the average monthly sunshine durations were 154.0, 176.5, and 200.2 h for 2016, 2017, and 2018, respectively (Supplementary Figure 2).

### Crop Cultivation and Treatments

The *japonica* super rice cultivar “Wuyunjing27” (WYJ27), a popular cultivar in this region, was selected. Seeds were sown in a nursing paddy and then grown under ambient air for 1 month. Seedlings were manually transplanted to all plots at a density of two seedlings per hill at around June 20 in each growing season. Nitrogen was applied in the form of urea (N, 46%) and compound chemical fertilizer (N:P_2_O_5_:K_2_O, 15:15:15) at a rate of 22.5 g N⋅m^–2^. In each growing season, nitrogen was split into three applications, with 40% of the total as a basal dressing 1 day before transplanting, 30% as a top dressing at the early tillering stage, and 30% as a top dressing at panicle ignition. Phosphorus and potassium were applied as compound fertilizer P_2_O_5_ and K_2_O at a rate of 9 g⋅m^–2^ 1 day before transplanting. The water regime was as follows: the paddy fields were submerged with water at a level of 5 cm from about June 17 to July 20 and subsequently subjected to wet-dry cycles through natural drainage and intermittent irrigation from about 21 July to 10 August. Diseases and insects were monitored and controlled during the growing seasons. Fertilizer application and water regime are described in detail elsewhere (Zhao et al., 2019). In each plot, 30 plants with similar tiller numbers were selected and labeled at heading; of these, 15 plants were subjected to leaf cutting (LC, cutting off top three leaves), and 15 plants were used as control crops (CK, no leaf cutting).

### Determination of Grain Quality Traits

At maturity, 10 plants were harvested from each treatment in each plot to determine quality. The grain was threshed manually, and fertile grains were selected using an airflow separator and placed in a natural environment for 3 months. Subsequently, the quality traits were determined according to GB/T 17891-2017 (General Administration of Quality Supervision, Inspection and Quarantine of the People’s Republic of China, 2018). The evaluation of rice processing quality included brown rice percentage (BRP), milled rice percentage (MRP), and head rice percentage (HRP); here, the head rice refers to the milled rice with a length greater than or equal to three-quarters of the whole kernel. These traits were calculated as follows:


BRP(%)=weightofbrownrice/sampleofroughrice×100%;

MRP(%)=weightofmilledrice/sampleofroughrice×100%;

HRP(%)=weightofheadrice/sampleofroughrice×100%.

The appearance characteristics of head rice were measured using a chalkiness visualization scanner equipped with JMWT12 software (Dongfujiuheng, Beijing, China). The appearance characteristic values included chalky grain percentage (CGP), chalkiness degree (CD), grain length, grain width, and the ratio of grain length to width.

After this, 50 g of brown rice and 50 g of milled rice were oven-dried at 60°C to constant weights and ground into powder by a vibration disk mill (TS1000, Siebtechnik GmbH, Mülheim, Germany) with a 100-mesh sieve to further prepare them for subsequent analyses.

The amylose concentration of milled rice was determined by iodine-blue colorimetry, based on the national standard GB/T 17891-2017. The starch viscosity of milled rice was determined using the rapid visco analyzer RVA-3D (Newport Scientific Inc., Warriewood, NSW, Australia), which was controlled by the thermo cycle for Windows (TCW). The experimental procedure was carried out using the American Association of Grain Chemists (AACC 1995-61-02) protocol. When the water content of rice flour was 12%, 3.0 g of milled rice powder and 25.0 g of distilled water were weighed. During the stirring process, the temperature in the tube changed as follows: below 50°C for 1 min, increasing to 95°C (over 3.8 min) at a rate of 11.8°C⋅min^–1^, and maintenance at 95°C for 1.4 min. The rotating speed of the agitator was 960 r⋅min^–1^ in the initial 10 s and then it was maintained at 160 r⋅min^–1^. The RVA characteristics included peak viscosity (PV), minimum viscosity (MV), final viscosity (FV), breakdown (BD: PV minus MV), setback (SB: FV minus PV), consistency (CS: FV minus MV), peak time (PT), and gelatinization temperature (GT).

The taste score of cooked rice was determined using a rice taste analyzer (STA-1A, SATAKE Co., Ltd., Hiroshima, Japan), to measure appearance, hardness, stickiness, balance degree, and overall palatability index (OPI); the first four indices received a maximum score of 10, and the OPI was evaluated with a full score of 100. The higher the score, the better the taste. The specific steps were as follows: we placed 30 g of milled rice into an aluminum jar, simulating the usual cooking process, washed it with a small amount of water, and then added water; the pot was weighed in advance to ensure that the ratio of rice to water was 1:1.3. The jar was covered with filter paper, tightly sealed with a rubber ring, and placed in an electric rice cooker for 30 min. After heating, we turned off the power, let the rice simmer in the pot for 10 min, removed it, slightly stirred it, and placed it into a cooling device for 10 min. After this, the rice was taken out and cooled at room temperature for 90 min, followed by placing it into a container until further analysis.

The nitrogen (N) concentrations of milled and brown rice were determined by an autoanalyzer (Kjeltec 8400, FOSS Analytical AB, Hillerød, Denmark) after hydrolysis by the Kjeldahl method; the results were multiplied by 5.95 to estimate the protein concentration. The mineral concentration was determined as follows. First, 0.50 g of flour was weighed in the lining tube of a microwave digestion apparatus (MARS5, CEM Corporation, Matthews, NC, United States), followed by the addition of 5 mL of 65% nitric acid, 3 mL of ultrapure water, and three drops of hydrogen peroxide. The mixture was then placed into a microwave digestion-meter for high-temperature digestion. After digestion, we removed the digestion tube, increased the volume of the digestion liquid to 50 mL, and then filtered it with quantitative filter paper. The mineral element concentrations of the filtrate were determined using an inductively coupled plasma emission spectrometer (iCAP6300, Thermo Fisher Scientific, Waltham, MA, United States); we determined the macroelements phosphorus (P), sulfur (S), potassium (K), calcium (Ca), and magnesium (Mg) and the microelements boron (B), copper (Cu), iron (Fe), manganese (Mn), and zinc (Zn).

The phytic acid concentration (PAC) of milled and brown rice was determined using the modified method of Vaintraub and Lapteva (1988). In brief, 0.25 g of the sample was weighed into a 10-mL tube, and 5 mL of 0.7% HCl was added to the tube for oscillation extraction (25°C, 150 r⋅min^–1^) for 1 h. After centrifugation (4,000 r⋅min^–1^, 15 min), the supernatant was placed into another tube, spiked with chromogenic reagent (FeCl_3_ and sulfosalicylic acid), mixed well, and again centrifuged (3,400 r⋅min^–1^, 10 min). The absorbance of the obtained supernatant was measured at 500 nm. The PAC was calculated according to a standard curve prepared using sodium phytate.

### Statistical Analysis

The field experiment followed a completely randomized design with a split-plot arrangement. The CO_2_ was treated as the main plot, and different source-sink treatments were treated as subplots with three replications. Analysis of variance (ANOVA) was performed using SPSS statistical software (SPSS 20.0, IBM company, Chicago, IL, United States). The least significant difference procedure was used to compare the means among treatments. Statistically significant effects were indicated as follows: ^∗∗^*P* < 0.01, ^∗^*P* < 0.05, ^+^*P* < 0.1. Pearson’s correlation coefficients were calculated to determine the relationships among the different parameters.

## Results

### Processing Quality

The processing quality of rice is usually expressed as the brown rice percentage (BRP), the milled rice percentage (MRP), and the head rice percentage (HRP). These three parameters are defined as the percentages of brown rice, milled rice, and complete milled rice (including complete milled rice and about 3/4 of the size of the whole milled rice) to the weight of the total rough grain. Based on the 3-year field experiment, the values of BRP, MRP, and HRP varied significantly among years. The BRP reached its maximum in 2017, whereas MRP and HRP were highest in 2018 (Table 1). Compared with ambient CO_2_, eCO_2_ significantly reduced BRP, MRP, and HRP by 0.5, 2.1, and 4.2%, on average, respectively. Crops under CK and LC showed similar responses to eCO_2_. Compared with CK, the LC treatment had no effect on processing quality, but there was a significant interaction between LC and year, which showed that LC slightly increased the three parameters in 2016 and decreased them uniformly in the following 2 years. No significant interactions between CO_2_ and year or LC for BRP, MRP, and HRP were observed.

**TABLE 1:** Brown rice percentage (BRP), milled rice percentage (MRP) and head rice percentage (HRP) of WYJ27 as affected by elevated CO_2_ under CK and LC (cutting off top three leaves) across three cropping seasons (2016-2018); AC and EC refer to ambient CO_2_ and elevated CO_2_, respectively.

Year	Treatment	CO_2_	BRP (%)	MRP (%)	HRP (%)
2016	CK	AC	85.2 ± 0.22	71.7 ± 0.33	68.8 ± 0.73
		EC	84.3 ± 0.48	69.3 ± 1.44	64.7 ± 2.61
		% change	−1.0	−3.4	−5.9
	LC	AC	85.5 ± 0.13	73.2 ± 0.81	71.7 ± 1.39
		EC	85.2 ± 0.38	71.9 ± 0.38	68.5 ± 0.72
		% change	−0.4	−1.8	−4.4
2017	CK	AC	85.2 ± 0.03	72.3 ± 0.70	69.0 ± 1.61
		EC	85.2 ± 0.31	71.0 ± 0.77	66.3 ± 1.39
		% change	0.0	−1.7	−3.9
	LC	AC	84.9 ± 0.46	71.7 ± 0.84	68.4 ± 1.47
		EC	84.4 ± 0.15	69.5 ± 0.58	64.5 ± 0.90
		% change	−0.7	−3.0	−5.8
2018	CK	AC	84.4 ± 0.24	73.2 ± 0.44	71.0 ± 0.76
		EC	84.0 ± 0.17	72.2 ± 0.53	69.5 ± 0.56
		% change	−0.5	−1.3	−2.1
	LC	AC	84.1 ± 0.31	72.6 ± 0.40	69.8 ± 0.65
		EC	83.9 ± 0.17	71.7 ± 0.47	67.6 ± 1.56
		% change	−0.2	−1.2	−3.2
ANOVA					
Year			**	*	0.051
CO_2_			*↓	**↓	**↓
LC			ns	ns	ns
CO_2_ × Year			ns	ns	ns
CO_2_ × LC			ns	ns	ns
LC × Year			*	*	*
CO_2_ × LC × Year			ns	ns	ns

Arrows in the treatment column indicate treatment decreased (↓) the values. Values are means ± standard error (n = 3); Statistically significant effects are indicated as **P < 0.01; *P < 0.05. The value indicates the probability between 0.05 and 0.1. ns, not significant.

### Appearance Quality

**FIGURE 1:**
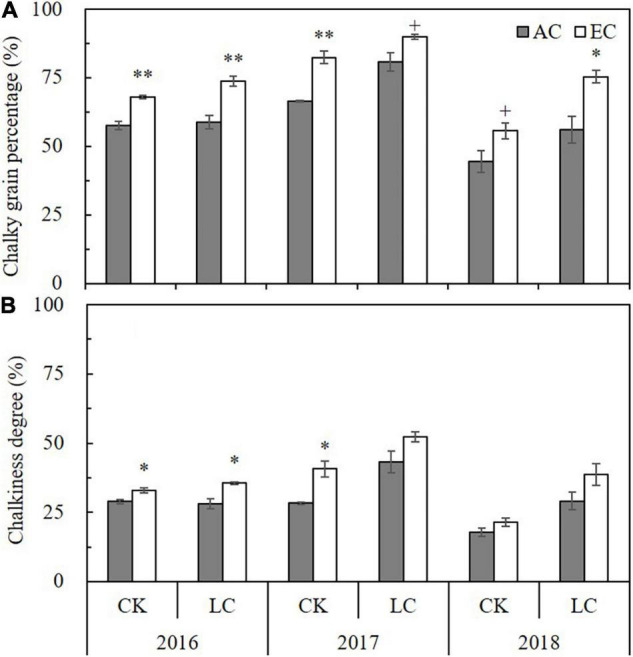
Chalky grain percentage **(A)** and chalkiness degree **(B)** of WYJ27 as affected by elevated CO_2_ under CK (no leaf cutting) and LC (cutting off top three leaves) across three cropping seasons (2016–2018). Each bar in the figure represents the mean values across three plots for ambient CO_2_ (AC, filled square) or elevated CO_2_ (EC, AC + 200 ppm, unfilled square); vertical bars represent standard error (*n* = 3). Statistically significant effects are indicated as ^∗∗^*P* < 0.01; ^∗^*P* < 0.05; + *P* < 0.1; ns, not significant.

Chalky grain percentage (CGP) refers to the ratio of the number of chalky rice grains to the total number of rice grains, and chalkiness degree (CD) refers to the percentage of the chalky area of chalky rice to the total area of rice grains; both are important parameters indicating the appearance quality of rice. The CGP and CD values of milled rice significantly differed among the different growing seasons, such that those of 2017 were highest, following by those of 2016 and, lastly, 2018. Compared with ambient CO_2_, eCO_2_ significantly increased CGP and CD by 22.3% and 26.3%, on average, respectively, and CK and LC crops showed the same increasing trend (Figure 1). Compared with CK, averaged across 3 years, LC significantly increased CGP and CD by 16.2% and 33.2%, respectively; were highest in 2018, followed by 2017 and, lastly, 2016, which resulted in a significant LC by year interaction (Supplementary Table 1), whereas no significant interactions among other principal factors were observed.

The values of length, width, and length-to-width ratio of milled rice varied significantly among years (Table 2). In 2018, grain length and length-to-width ratio were both greater than those of the two previous seasons, but grain width was the smallest. Compared with ambient, eCO_2_ had no effect on grain width, but significantly increased grain length and length-to-width ratio by 1.1% and 1.2%, respectively, with both exhibiting the most significant increase in 2016, which led a significant CO_2_ by year interaction. Compared with CK, LC had no significant effect on grain shape.

**TABLE 2:** Effect of elevated CO_2_ and cutting off top three leaves (LC) on grain length, grain width, length-width ratio of milled rice across three cropping seasons (2016–2018).

Year	Treatment	CO_2_	Grain length (mm)	Grain width (mm)	Length-width ratio
2016	CK	AC	4.61 ± 0.07	2.66 ± 0.02	1.73 ± 0.02
		EC	4.81 ± 0.01	2.67 ± 0.01	1.80 ± 0.01
		% change	4.19	0.25	3.85
	LC	AC	4.67 ± 0.05	2.66 ± 0.01	1.76 ± 0.02
		EC	4.88 ± 0.02	2.69 ± 0.01	1.82 ± 0.01
		% change	4.57	1.13	3.42
2017	CK	AC	4.77 ± 0.01	2.68 ± 0.02	1.78 ± 0.02
		EC	4.72 ± 0.02	2.66 ± 0.01	1.78 ± 0.01
		% change	−0.98	−0.75	−0.19
	LC	AC	4.78 ± 0.04	2.69 ± 0.01	1.78 ± 0.02
		EC	4.71 ± 0.05	2.67 ± 0.01	1.76 ± 0.01
		% change	−1.60	−0.87	−0.75
2018	CK	AC	4.81 ± 0.04	2.60 ± 0.01	1.85 ± 0.02
		EC	4.82 ± 0.04	2.59 ± 0.03	1.86 ± 0.01
		% change	0.21	−0.38	0.54
	LC	AC	4.83 ± 0.06	2.62 ± 0.01	1.85 ± 0.02
		EC	4.86 ± 0.08	2.63 ± 0.04	1.85 ± 0.01
		% change	0.62	0.25	0.18
ANOVA results					
Year			*	**	**
CO_2_			*↑	ns	*↑
LC			ns	ns	ns
CO_2_ × Year			**	ns	**
CO_2_ × LC			ns	ns	ns
LC × Year			ns	ns	ns
CO_2_ × LC × Year			ns	ns	ns

AC and EC refer to ambient CO_2_ and elevated CO_2_, respectively. Arrows in the treatment column indicate treatment increased (↑) the values. Values are means ± standard error (n = 3). Statistically significant effects are indicated as **P < 0.01; *P < 0.05; ns, not significant.

### Eating and Cooking Quality

**FIGURE 2:**
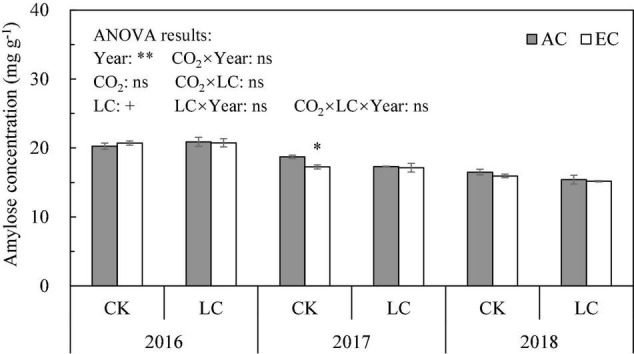
The amylose concentration of WYJ27 as affected by elevated CO_2_ under CK (no leaf cutting) and LC (cutting off top three leaves) across three cropping seasons (2016–2018). Each bar in the figure represents the mean values across three plots for ambient CO_2_ (AC, filled square) or elevated CO_2_ (EC, AC + 200 ppm, unfilled square); vertical bars represent standard error (*n* = 3). Statistically significant effects are indicated as ^∗∗^*P* < 0.01; ^∗^*P* < 0.05; + *P* < 0.1; ns, not significant.

The amylose concentration of milled rice was significantly affected by the growing seasons, with the highest concentration observed in 2016, followed by 2017 and, lastly, 2018 (Figure 2). Compared with ambient CO_2_, eCO_2_ reduced amylose concentration by 1.9%, on average, across three growing seasons, and reduced amylose concentration of CK and LC crops by 2.8 and 1.0%, respectively, with CK crops showing a significant difference (*P* < 0.1). Compared with CK, LC reduced amylose concentration by an average of 2.5% (*P* < 0.1). No significant interactions between year and CO_2_ or LC for amylose concentration were observed.

The RVA-value represents the viscosity curve and disintegration of starch along with temperature. The values of RVA parameters, except PT and GT, varied significantly among years (Table 3), such that the highest values were observed in 2016, followed by 2017 and, lastly, 2018. Compared with ambient, eCO_2_ significantly increased peak viscosity (PV) and minimum viscosity (MV) by 2.9% and 3.8%, respectively, whereas setback (SB) and consistency (CS) significantly decreased by 96.1% and 4.7%, respectively, with no significant effects on breakdown (BD), final viscosity (FV), peak time (PT), or gelatinization temperature (GT). Regarding crops under different source-sink ratios, for CK crops, eCO_2_ increased PV, MV, PT, and GT (by less than 3.0%) and decreased SB and CS by 76.7% and 5.2% (*P* < 0.1), respectively, on average; for LC crops, eCO_2_ increased PV and MV by 4.1% (*P* < 0.05) and 4.6% (*P* < 0.1), respectively, and decreased SB and CS by 118.9% (*P* < 0.01) and 4.1%, respectively. Compared with CK, LC reduced SB and CS by 14.9% and 5.0% (*P* < 0.05), respectively, and increased GT by 2.2% on average. Among the three seasons, the effects of LC treatment on PV, MV, SB, and CS were greatest in 2018, which resulted in a significant LC by year interaction.

**TABLE 3:** Effect of elevated CO_2_ and cutting off top three leaves (LC) on RVA characteristics of milled rice across three cropping seasons (2016–2018).

Year	Treatment	CO_2_	Peak viscosity (cP)	Minimum viscosity (cP)	Breakdown (cP)	Final viscosity (cP)	Setback (cP)	Consistence (cP)	Peak time (min)	Gelatinization temperature (°C)
2016	CK	AC	3927.3 ± 29.8	2455.3 ± 22.9	1472.0 ± 20.4	3817.3 ± 18.8	−110.0 ± 11.1	1362.0 ± 14.7	6.36 ± 0.06	74.20 ± 0.28
		EC	3934.7 ± 86.5	2472.3 ± 56.8	1462.3 ± 30.0	3816.7 ± 68.0	−118.0 ± 18.9	1344.3 ± 13.0	6.40 ± 0.04	73.90 ± 0.28
		% change	0.19	0.69	−0.66	−0.02	−7.27	−1.30	0.70	−0.40
	LC	AC	3748.3 ± 35.3	2367.3 ± 49.7	1381.0 ± 14.7	3735.3 ± 43.5	−13.0 ± 10.7	1368.0 ± 7.5	6.27 ± 0.08	82.28 ± 3.92
		EC	3877.0 ± 110.7	2421.3 ± 84.4	1455.7 ± 39.2	3777.3 ± 95.5	−99.7 ± 24.7	1356.0 ± 15.0	6.29 ± 0.06	74.95 ± 0.50
		% change	3.43	2.28	5.41	1.12	−666.67	−0.88	0.35	−8.91
2017	CK	AC	3763.7 ± 67.2	2365.3 ± 35.1	1398.3 ± 32.3	3696.0 ± 63.9	−67.7 ± 5.8	1330.7 ± 29.5	6.33 ± 0.04	72.78 ± 0.02
		EC	3785.3 ± 27.0	2391.7 ± 51.2	1393.7 ± 62.3	3621.7 ± 52.2	−163.7 ± 68.3	1230.0 ± 13.11	6.36 ± 0.06	73.92 ± 0.27
		% change	0.58	1.11	−0.33	−2.01	−141.87	−7.57	0.35	1.56
	LC	AC	3630.0 ± 57.6	2309.3 ± 36.1	1320.7 ± 23.5	3566.0 ± 58.5	−64.0 ± 1.5	1256.7 ± 24.8	6.36 ± 0.02	73.62 ± 0.02
		EC	3851.7 ± 61.8	2437.7 ± 28.5	1414.0 ± 33.5	3625.0 ± 31.5	−226.7 ± 55.5	1187.3 ± 29.3	6.38 ± 0.02	73.42 ± 0.74
		% change	6.11	5.56	7.07	1.65	−254.17	−5.52	0.35	−0.27
2018	CK	AC	3431.3 ± 21.6	2026.7 ± 94.4	1404.7 ± 110.7	3353.3 ± 48.4	−78.0 ± 63.5	1326.7 ± 47.2	6.24 ± 0.12	75.82 ± 0.70
		EC	3584.7 ± 64.9	2186.3 ± 93.8	1398.3 ± 151.5	3422.3 ± 9.3	−162.3 ± 62.9	1236.0 ± 91.3	6.36 ± 0.09	77.05 ± 0.28
		% change	4.47	7.88	−0.45	2.06	−108.12	−6.83	1.82	1.63
	LC	AC	3606.3 ± 47.7	2256.7 ± 103.4	1349.7 ± 66.4	3431.3 ± 88.7	−175.0 ± 47.3	1174.7 ± 83.3	6.24 ± 0.12	76.25 ± 0.28
		EC	3715.0 ± 100.4	2390.7 ± 93.1	1324.3 ± 7.5	3489.7 ± 131.0	−225.3 ± 33.6	1099.0 ± 41.0	6.40 ± 0.07	77.02 ± 0.29
		% change	3.01	5.94	−1.88	1.70	−28.76	–6.44	2.56	1.01
ANOVA										
Year			**	**	ns	**	0.051	**	ns	**
CO_2_			**↑	*↑	ns	ns	**↓	*↓	ns	ns
LC			ns	ns	ns	ns	ns	*↓	ns	*↑
CO_2_ × Year			ns	ns	ns	ns	ns	ns	ns	*
CO_2_ × LC			ns	ns	ns	ns	ns	ns	ns	*
LC × Year			*	*	ns	ns	0.077	0.060	ns	*
CO_2_ × LC × Year			ns	ns	ns	ns	ns	ns	ns	ns

AC and EC refer to ambient CO_2_ and elevated CO_2_, respectively. Arrows in the treatment column indicate treatment increased (↑) or decreased (↓) the values. Values are means ± standard error (n = 3). Statistically significant effects are indicated as **P < 0.01; *P < 0.05. The value indicates the probability between 0.05 and 0.1. ns, not significant.

The rice taste analyzer can directly evaluate the quality of cooked rice. The rice taste parameters varied significantly among years (Table 4), with the values for appearance, stickiness, balance degree, and overall palatability index (OPI) being highest in 2017, whereas hardness was lowest in 2017. Compared with ambient CO_2_, eCO_2_ increased rice appearance, stickiness, balance, and OPI by 4.5% (*P* < 0.01), 3.5% (*P* < 0.1), 4.8% (*P* < 0.01), and 4.0% (*P* < 0.05), respectively, and significantly reduced hardness by 6.7%. The response of LC crops to eCO_2_ was similar to that of CK crops. Compared with CK, LC had no significant effect on cooked rice parameters, but the influence of LC in 2016 and 2017 was greater than that in 2018, resulting in a significant LC by year interaction, whereas no significant interaction among other main factors was observed.

**TABLE 4:** Effect of elevated CO_2_ and cutting off top three leaves (LC) on the taste of cooked milled rice across three cropping seasons (2016–2018).

Year	Treatment	CO_2_	Appearance	Hardness	Stickiness	Balance degree	Overall palatability index
2016	CK	AC	8.67 ± 0.08	5.43 ± 0.08	9.27 ± 0.10	9.01 ± 0.09	83.24 ± 1.64
		EC	8.91 ± 0.04	5.00 ± 0.05	9.45 ± 0.06	9.32 ± 0.05	85.50 ± 0.25
		% change	2.71	−7.84	1.94	3.47	2.71
	LC	AC	8.09 ± 0.29	5.86 ± 0.20	8.81 ± 0.19	8.42 ± 0.29	79.12 ± 2.59
		EC	8.41 ± 0.33	5.42 ± 0.18	8.94 ± 0.35	8.78 ± 0.33	80.79 ± 3.36
		% change	3.98	−7.45	1.46	4.32	2.10
2017	CK	AC	7.71 ± 0.10	6.06 ± 0.05	8.42 ± 0.14	8.06 ± 0.11	75.17 ± 1.20
		EC	8.45 ± 0.15	5.60 ± 0.03	9.04 ± 0.18	8.77 ± 0.12	81.42 ± 2.02
		% change	9.62	−7.57	7.43	8.79	8.31
	LC	AC	8.18 ± 0.15	5.80 ± 0.06	8.50 ± 0.24	8.53 ± 0.12	79.08 ± 1.58
		EC	8.70 ± 0.12	5.46 ± 0.21	9.01 ± 0.41	9.04 ± 0.16	84.92 ± 1.04
		% change	6.42	−5.89	5.98	5.96	7.38
2018	CK	AC	8.45 ± 0.08	5.63 ± 0.15	9.17 ± 0.07	9.63 ± 0.13	97.00 ± 0.58
		EC	8.54 ± 0.16	5.23 ± 0.12	9.38 ± 0.10	9.65 ± 0.20	97.92 ± 0.08
		% change	1.10	−7.25	2.36	0.17	0.95
	LC	AC	8.36 ± 0.19	5.43 ± 0.21	8.99 ± 0.42	9.05 ± 0.20	92.33 ± 4.59
		EC	8.65 ± 0.19	5.22 ± 0.08	9.17 ± 0.61	9.69 ± 0.10	95.83 ± 2.17
		% change	3.41	−3.99	1.95	7.00	3.79
ANOVA							
Year			0.097	**	0.093	**	**
CO_2_			**↑	**↓	0.082↑	**↑	*↑
LC			ns	ns	ns	ns	ns
CO_2_ × Year			ns	ns	ns	ns	ns
CO_2_ × LC			ns	ns	ns	ns	ns
LC × Year			**	**	ns	**	*
CO_2_ × LC × Year			ns	ns	ns	ns	ns

AC and EC refer to ambient CO_2_ and elevated CO_2_, respectively. Arrows in the treatment column indicate treatment increased (↑) or decreased (↓) the values. Values are means ± standard error (n = 3). Statistically significant effects are indicated as **P < 0.01; *P < 0.05. The value indicates the probability between 0.05 and 0.1; ns, not significant.

### Nutritional Quality

**FIGURE 3:**
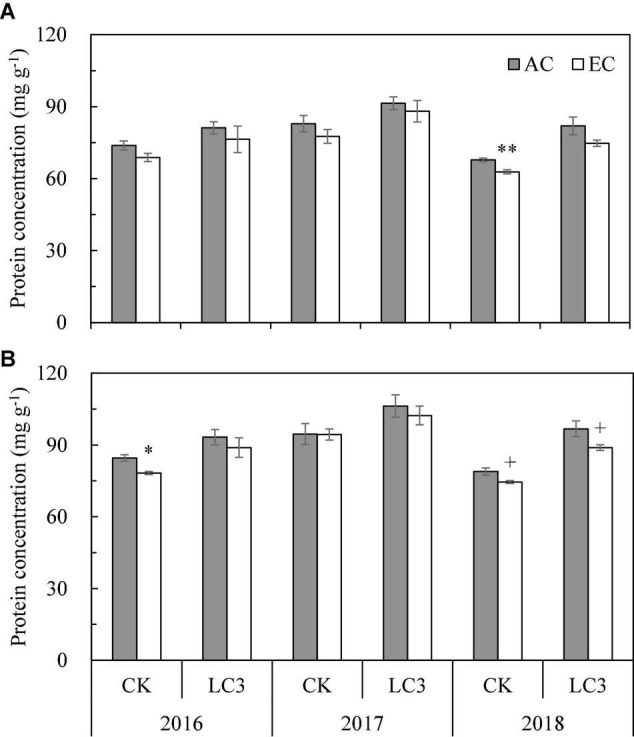
The protein concentration in milled rice **(A)** and brown rice **(B)** of WYJ27 as affected by elevated CO_2_ under CK (no leaf cutting) and LC (cutting off top three leaves) across three cropping seasons (2016–2018). Each bar in the figure represents the mean values across three plots for ambient CO_2_ (AC, filled square) or elevated CO_2_ (EC, AC + 200 ppm, unfilled square); vertical bars represent standard error (*n* = 3). Statistically significant effects are indicated as ^∗∗^*P* < 0.01; ^∗^*P* < 0.05; + *P* < 0.1.

Nutritional quality is directly related to the intake of grain protein and mineral elements. A significant year effect was observed for protein concentration (PC) in milled and brown rice (Supplementary Tables 2, 3), such that it was highest in 2017, followed by 2016 and, lastly, 2018 (Figure 3). Averaged across 3 years, PC in milled rice (77.3 mg⋅g^–1^) was significantly lower than that in brown rice (90.1 mg⋅g^–1^). Compared with ambient CO_2_, eCO_2_ significantly reduced PC by 5.3% on average, irrespective of the growing season, position, or LC treatment. Compared with CK, LC significantly increased PC by 14.6% on average, with 13.9% in milled rice and 15.1% in brown rice. No significant interaction was found among year, CO_2_, LC, and position on PC (Supplementary Table 4).

**FIGURE 4:**
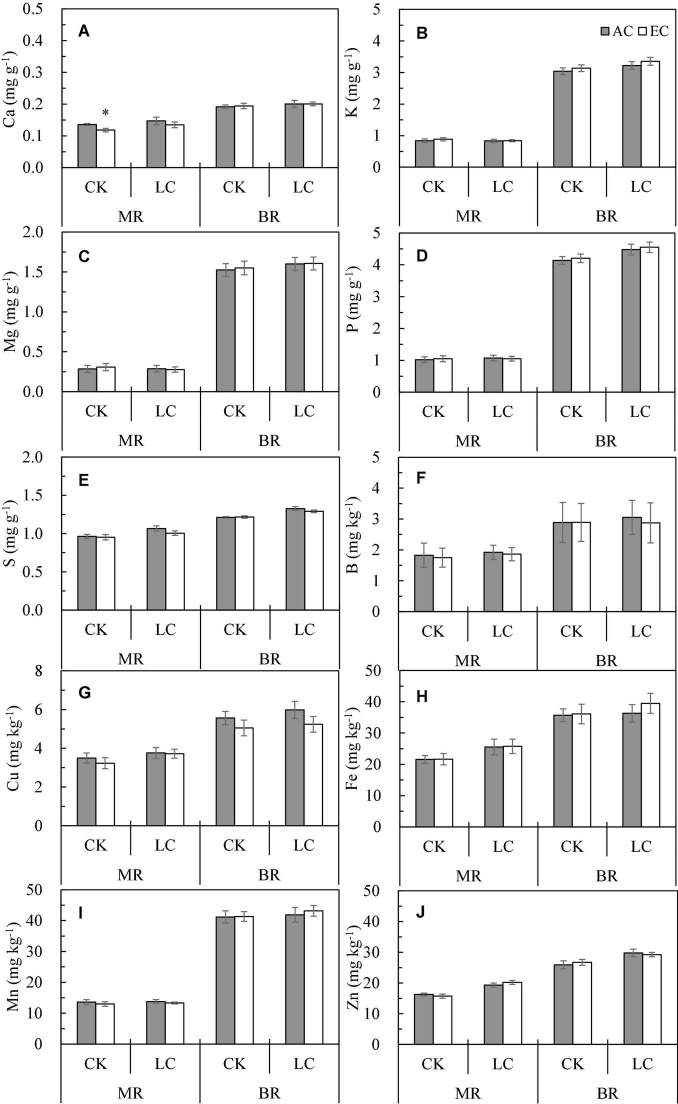
The concentration of Ca **(A)**, K **(B)**, Mg **(C)**, P **(D)**, S **(E)**, B **(F)**, Cu **(G)**, Fe **(H)**, Mn **(I)**, Zn **(J)** in milled rice (MR) and brown rice (BR) of WYJ27 as affected by elevated CO_2_ under CK (no leaf cutting) and LC (cutting off top three leaves) across three cropping seasons (2016–2018). Each bar in the figure represents the mean values across three plots for ambient CO_2_ (AC, filled square) or elevated CO_2_ (EC, AC + 200 ppm, unfilled square); vertical bars represent standard error (*n* = 3). Statistically significant effects are indicated as ^∗^*P* < 0.05.

Based on our results, the concentrations of macroelements Ca, K, Mg, P, and S and microelements B, Cu, Fe, Mn, and Zn in rice varied significantly among years. The levels of macroelements were lowest in 2016, whereas those of microelements were lowest in 2016 or 2018. Averaged across the 3 years, the mineral concentrations in brown rice were significantly higher than those in milled rice, with the levels of K, Mg, P, and Mn in brown rice being 2–5 times higher than those in milled rice (Figure 4). We observed a significant interaction between year and position for elements except Ca. Compared with ambient CO_2_, eCO_2_ significantly reduced Ca, S, and Cu concentrations by 4.7, 2.2, and 9.6%, respectively, and increased K by 3.9% on average, without any effects on other elements. Further analysis of Cu concentrations for different positions and leaf cutting treatments showed that eCO_2_ significantly reduced the Cu concentration in brown rice by 13.2%, but no significant change was observed in milled rice. Further, Cu was decreased by 11.8% (*P* = 0.107) and 7.4% (*P* = 0.087) in CK and LC crops, respectively. Compared with CK, LC had no significant effects on B and Mn, but increased Ca, K, Mg, P, S, Cu, Fe, and Zn concentrations by 2–16%. Significant interactions between LC and position for K, Mg, and P and also significant interactions between LC and year for Ca, K, and B were observed.

**FIGURE 5:**
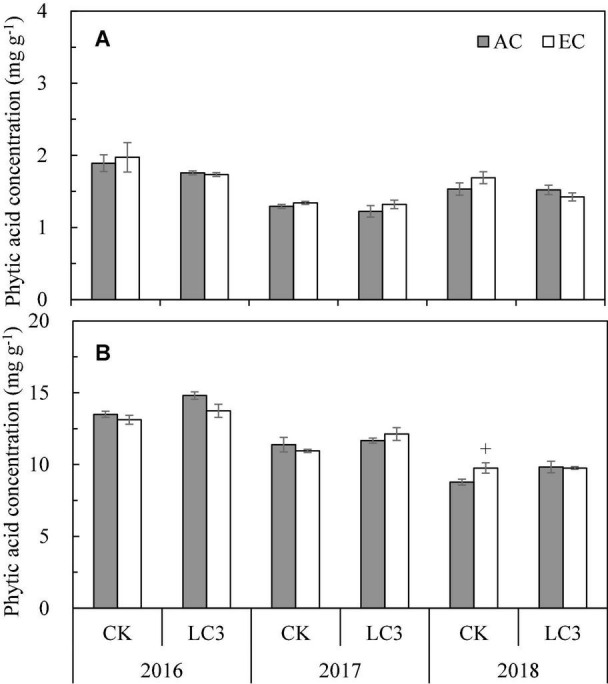
The phytic acid concentration in milled rice **(A)** and brown rice **(B)** of WYJ27 as affected by elevated CO_2_ under CK (no leaf cutting) and LC (cutting off top three leaves) across three cropping seasons (2016-2018). Each bar in the figure represents the mean values across three plots for ambient CO_2_ (AC, filled square) or elevated CO_2_ (EC, AC + 200 ppm, unfilled square); vertical bars represent standard error (*n* = 3). Statistically significant effects are indicated as + *P* < 0.1.

The phytic acid concentration (PAC) of rice varied significantly among years, with the highest value observed in 2016, followed by 2017 and, lastly, 2018. Averaged across all treatments, PAC in milled rice and brown rice was 1.56 and 11.62 mg⋅g^–1^, respectively, with a significant difference between the two positions (*P* < 0.01) (Figure 5). Compared to ambient CO_2_, eCO_2_ increased PAC by 2.4% on average, but not significantly (*P* = 0.841), which is mainly owing to a large increase in 2018. Under eCO_2_, the increase rates of PAC in milled rice and brown rice were similar, and leaf cutting had no significant effect on the response of PAC to eCO_2_, which resulted in a significant CO_2_ by year interaction, whereas no significant interaction between CO_2_ and LC was observed. Compared with CK, LC significantly increased the PAC by 4.3% on average, which was mainly related to the larger increase in brown rice (+ 6.2%, *P* < 0.01), resulting in a significant LC by position interaction (Supplementary Table 4).

## Discussion

### Processing Quality

Our 3-year FACE study showed that eCO_2_ significantly reduced brown rice percentage (BRP) and milled rice percentage (MRP) by 1–2% on average. Although this decrease was small, it was statistically significant (Table 1). This is consistent with the findings of previous FACE studies (Yang et al., 2007; Wang et al., 2011), indicating that rice grown under eCO_2_ loses more glume or aleurone layers during processing compared to rice cultivated under ambient CO_2_. Compared with BRP and MRP, the HRP decreased more significantly (4.2%) under eCO_2_, which was consistent with the findings of previous FACE experiments (Yang et al., 2007; Jing et al., 2016a; Niu et al., 2021); these results reflect that rice cultivated under eCO_2_ is more sensitive to processing procedures. Xu et al. (2008) speculated that decreases in BRP and MRP under eCO_2_ may be caused by the accumulation of a large amount of assimilation products in the glume or aleurone layers of rice under FACE conditions, but it is still necessary to further clarify this by studying the grain-filling process specifically. High temperatures may also be one of the factors that lead to the decrease in HRP under eCO_2_. A FACE study by Zhang et al. (2015) showed that the partial closure of the stoma in rice leaves under eCO_2_ can lead to increased panicle temperatures. We did not measure canopy or panicle temperatures in this study, but previous rice FACE studies reported a 0.43°C increase in canopy temperature averaged from anthesis to maturity (Luo et al., 2002) and a 1–2°C increase in leaf temperature at the flowering stage (Yoshimoto et al., 2005) under eCO_2_, which likely also occurred in our FACE experiment. When ambient temperature was already high, the probability of further grain chalkiness increase by a less than 1°C elevation in temperature (from warming effect of FACE) was also high, especially for sensitive cultivars. The chalky grain percentage of the *japonica* cultivar used in this study already reached 50% in ambient plots (Figure 1). High temperatures can also affect the activity of starch synthase (Cheng et al., 2005), resulting in the formation of space in the endosperm, which makes the grains brittle.

Unlike increasing source (eCO_2_), the treatment of reducing source (LC) had no significant effect on the processing quality of rice. This is in agreement with the findings of Yuan et al. (2005); according to these authors, BRP, MRP, and HRP were significantly reduced when all the green leaves were cut off, indicating that the effect of leaf cutting treatment on processing quality was related to the degree of leaf cutting. In the present study, there was no interaction effect between CO_2_ and LC on the above three parameters, indicating that leaf cutting did not change the response of rice processing quality to eCO_2_.

### Appearance Quality

**FIGURE 6:**
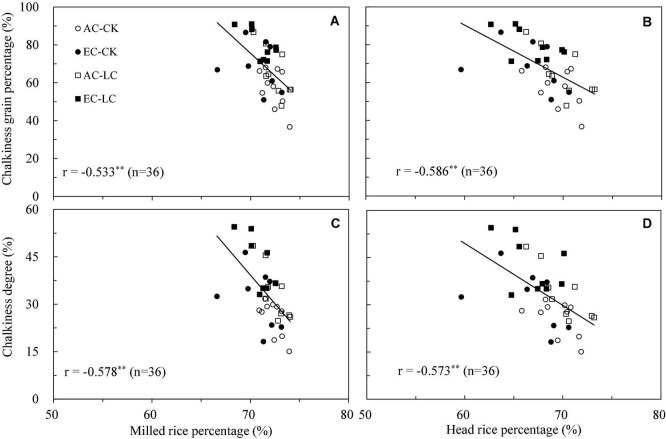
Relationship between chalkiness grain percentage and milled rice percentage **(A)**, chalkiness grain percentage and head rice percentage **(B)**, chalkiness degree and milled rice percentage **(C)**, chalkiness degree and head rice percentage **(D)** for each combination of year × CO_2_ × LC × plot in 2016-2018. AC, ambient CO_2_; EC, elevated CO_2_ (AC + 200 ppm). CK, no leaf cutting; LC, cutting off top three leaves. Statistically significant effects are indicated as ^∗∗^*P* < 0.01.

As indicated in a review by Wang and Yang (2020), the grain size of rice grown in an eCO_2_ environment increases or decreases only slightly. However, chalkiness mostly increases. The present study found that rice width did not change under eCO_2_, whereas length and length-width ratio increased slightly (by about 1%). As opposed to grain size, FACE treatment significantly increased the chalkiness of WYJ27: the average CGP and CD values increased by 22% and 26%, respectively, under different leaf cutting treatments across the three growing seasons. The increase in chalkiness is obviously smaller than that reported in previous studies using the cultivar “Wuyunjing 23” (> 50%, Jing et al., 2016a, 2021b; Hu et al., 2019) but greater than or close to the values from other reports (Yang et al., 2007; Wang et al., 2014; Niu et al., 2021). These results indicate that the chalkiness of the Wuyunjing series of cultivars, especially the early varieties, may be more sensitive to eCO_2_. The increased chalkiness of FACE rice is associated with loosely stacked starch in grains, which are easily broken into pieces during processing. This result is consistent with the decline in the processing quality under FACE conditions (Table 1). There was a significant negative correlation between MRP and CGP (*r* = −0.553^∗∗^, Figure 6A) and MRP and CD (*r* = −0.578^∗∗^, Figure 6C), as well as a significant negative correlation between HRP and CGP (*r* = -0.586∗^∗^, Figure 6B) and HRP and CD (*r* = −0.573^∗∗^, Figure 6D). Similar results have been reported by Dong et al. (2004) and Yang et al. (2007). The increased chalkiness under eCO_2_ is mainly related to the high spike temperature caused by the partial closure of leaf stoma, accompanied by rapid grain-filling in the early stage and early senescence in the later stage of grain-filling (Yang et al., 2007; Wang and Yang, 2020). Previous studies have shown that both elevated CO_2_ and temperature increased grain chalkiness, and the combination of the two treatments further increased chalky grain percentage and chalkiness degree (Jing et al., 2016a; Usui et al., 2016). Although grain filling parameters were not measured in this study, the growth period survey showed that the maturation of rice in the FACE plot occurred 2 days earlier than that in the ambient CO_2_ plot (Supplementary Figure 1), which shortened the duration of grain filling.

Similar to eCO_2_, LC significantly increased the chalkiness of rice. Averaged across the 3 years, LC significantly increased CGP and CD by 16% and 33%, respectively. This was consistent with previous artificial source reduction studies on leaf cutting and shading (Yuan et al., 2005; Tao et al., 2006) and may be associated with the reduction of the supply of assimilate compounds caused by artificial source reduction (leaf cutting or shading). Both eCO_2_ (source stimulation) and LC (source reduction) increased grain chalkiness; thus, they seem to have different physiological causes. A meta-analysis of 20 years of FACE studies showed that eCO_2_ enhances rice yield mainly through the increase of spikelet density (Hu et al., 2021); therefore, the eCO_2_ applied throughout the crop cycle increases not only the source (photosynthesis) but also the sink (panicle and spikelet number). Source-sink balance may constantly shift during plant growth. Because of the acceleration of senescence of leaves or whole plants under FACE (Huang et al., 2005; Shimono et al., 2010), there was a shortening of grain-filling duration that consequently enhanced source limitation at the later stage of grain filling, which was similar to the effect of LC on source reduction. In contrast, removing spikelets usually significantly reduces rice chalkiness (Tao et al., 2006; Jing et al., 2016b). Previous studies have shown that the response of grain chalkiness to eCO_2_ may be affected by the cultivar (Wang et al., 2019; Niu et al., 2021) or the location of the grain on the panicle (Hu et al., 2019), whether this response is affected by source reduction (LC) remains unknown. The present study shows that reducing the source strength by LC at the heading stage did not affect the effect of eCO_2_ on chalkiness as there was no interaction effect between CO_2_ and LC (Supplementary Table 1). As shown in our previous FACE study, removing spikelets at the heading stage also does not change the responses of CGP or CD of “Wuyunjing 23” to eCO_2_ (Jing et al., 2016b). This indicates that changing the source-sink ratio at the heading stage cannot change the response of chalkiness to eCO_2_; however, this may need to be verified with the use of more different genotypes.

### Eating and Cooking Quality

High-quality rice is gaining increased popularity among consumers. The amylose concentration is often used as one of the indicators of rice eating quality (Jing et al., 2016a). In this study, overall, eCO_2_ decreased the amylose concentration in milled rice (by about 2%), although not significantly; there was no significant interaction between CO_2_ and LC or year (Figure 2). In previous studies, amylose concentration increased (Seneweera and Conroy, 1997; Hu et al., 2019), decreased (Yang et al., 2007; Goufo et al., 2014) or remained unchanged (Terao et al., 2005; Jing et al., 2016b, 2021a) under eCO_2_, indicating that the influence of eCO_2_ on amylose concentration in rice is inconsistent. The RVA characteristic value is often used to represent rice eating quality. In this study, FACE treatment significantly increased the PV and MV values of milled rice and significantly decreased SB and CS. The changes in these parameters were consistent with the findings of previous studies (Wang et al., 2014; Jing et al., 2016a), indicating that eCO_2_ can improve rice eating quality (Allahgholipour et al., 2006).

The palatability of rice is an intuitive reflection of its eating quality. However, few studies have directly evaluated the effect of eCO_2_ on cooked rice. Compared with traditional sensory evaluation, the rice taste analyzer can avoid bias caused by age, gender, personal preference, and other factors, resulting in greater objectivity. The taste analyzer results showed that eCO_2_ significantly increased the OPI of rice by 4% on average, which was reflected in the significant increase in appearance, stickiness, balance degree, and decrease of hardness (Table 4), indicating that eCO_2_ significantly improved the cooking and eating quality of rice. This is consistent with the results of the RVA spectrum and in agreement with the findings of Jing et al. (2016a,2021a). The increase in rice eating quality value under eCO_2_ has been associated with a decrease in amylose concentration and protein concentration (Cameron and Wang, 2005; Mohapatra and Bal, 2006; Jing et al., 2016a, 2021b), which is also confirmed by the results of this study (Figures 2, 3). Correlation analysis also showed that OPI was significantly negatively correlated with protein concentration (*r* = −0.626^∗∗^) and amylose concentration (*r* = −0.673^∗∗^), and protein concentration was significantly negatively correlated with stickiness (*r* = −0.793^∗∗^) and balance degree (*r* = −0.695^∗^) and positively correlated with hardness (*r* = 0.579^∗^, Supplementary Table 5). In addition, the cooking quality of rice may also be related to changes in the starch structure. According to the latest FACE study by our research group, eCO_2_ can cause the generation of large starch granules and increase cohesiveness of the starch structure (Jing et al., 2021a,b), thereby improving rice palatability.

Compared with CK, LC reduced the amylose concentration of milled rice by 2.5% (*P* < 0.1), but it had little influence on the parameters of the RVA spectrum or of cooked rice. Yuan et al. (2005) also observed a decreasing trend of the amylose concentration when reducing the source-sink ratio *via* cutting leaves or shading, indicating that artificial source reduction to reduce the supply of assimilates may reduce amylose synthesis. They also reported that the effects of leaf cutting on some RVA parameters (such as PV, BD, and SB) differed among different rice varieties, indicating that the effect of LC on RVA may be related to the tested varieties. Overall, eCO_2_ had a significantly greater effect on rice eating quality than the LC treatment.

### Nutritional Quality

In the present study, the rice protein concentration (PC) decreased by about 5% under eCO_2_ across the three seasons, which is in agreement with previous studies (Yang et al., 2007; Myers et al., 2014; Zhang et al., 2015); this phenomenon was not affected by CO_2_ fumigation (Taub et al., 2008). Zhu et al. (2018) cultivated 18 rice varieties of a wide genotypic and phenotypic range and found that the PC of all varieties showed a decreasing trend under FACE eCO_2_ treatment; the average decrease value was twice that of our study. We also show that eCO_2_ significantly reduced the concentrations of Ca, S, and Cu in rice, which has also been observed elsewhere (Yang et al., 2007; Loladze, 2014; Ujiie et al., 2019). Some studies have pointed out that the decrease in grain PC may be owing to the fact that eCO_2_ reduces the transpiration rate of leaves and the N requirement, thus reducing the N absorption rate of roots (Conroy and Hocking, 1993). According to some studies, the effect of eCO_2_ on the PC of rice can be explained by the increased photosynthetic rate and carbohydrate accumulation, causing a “dilution effect” on the N concentration (O’Neill et al., 1987; Gifford et al., 2000; Uprety et al., 2010; Panozzo et al., 2014; Chaturvedi et al., 2017); this effect has also been considered the main reason for the decrease in the mineral concentration in rice under eCO_2_ (Loladze, 2014). However, except for the significant decrease in N concentration, other elements such as P, K, Mg, S, and Zn, were not significantly impacted by eCO_2_, and Zn and Mn even showed a strong increasing trend (Lieffering et al., 2004). We also found that eCO_2_ increased the concentrations of some elements (such as K), indicating that the “dilution effect” cannot fully explain the changes of elements under eCO_2_. Ujiie et al. (2019) pointed out that plant carbohydrate transport under eCO_2_ may affect the expression levels of genes related to mineral elements transport, thus affecting the absorption and/or transport of minerals. From this perspective, mineral changes under eCO_2_ need to be further investigated. In this study, protein and mineral element concentrations were higher in brown than in milled rice, which has also been observed previously (Jiang et al., 2018). Therefore, the consumption of brown rice is more beneficial to human health. Despite the differences between milled and brown rice, the responses of the nutritional parameters for the two positions to eCO_2_ were similar.

Compared with the CK treatment, the concentrations of protein and minerals increased under the LC treatment. According to Fangmeier et al. (1999), crops have completed the process of absorbing N from the soil at the flowering stage, and after flowering, all the N in the grains is derived from reallocation from vegetative organs. As reported by Sperotto et al. (2013), cutting off flag leaves increases the concentrations of Fe, Mn, and Zn in grains; these leaves may be preferential but not essential sources of minerals in grains. The elements supplied to the grain could be compensated for *via* remobilization from other leaves and stem sheathing or continuing uptake *via* the root system when cutting off the flag leaves. Therefore, leaf cutting at the heading stage may not affect the absorption of N in the early stage or the supply of elements to grains in the later stage. On the other hand, our previous study has shown that LC significantly reduced the percentage and weight of fully filled grains (Gao et al., 2021), which may also explain the increase in grain element concentrations in terms of a “concentration effect.” Although LC itself had effects on grain element concentrations, it had little effect on the response to CO_2_, and there was almost no interaction effect between LC and CO_2_ on the concentration of proteins or minerals, indicating that the nutrient quality of rice under eCO_2_ is only slightly affected by source reduction. Compared with ambient CO_2_, eCO_2_ also reduces the PC of both CK and spikelet removal crops (Jing et al., 2016b), and therefore, in terms of the source-sink relationship, leaf cutting or spikelet removal can change the supply and demand relationship of carbohydrates in grains, whereas it does not change the decreasing trend of PC under eCO_2_. This also suggests that the “dilution effect” cannot fully explain the decrease in mineral levels. However, rice may have more rapid digestibility because of the observed effects of eCO_2_ (loosely stacked starch, lower hardness, lower protein concentration, etc.), which has potential to increase the glycemic index and exacerbate the prevalence of obesity (Loladze, 2014).

Phytic acid is an antinutrient widely found in seeds and fruits, and it inhibits the intestinal absorption of essential minerals (Miller et al., 2007; Schlemmer et al., 2009). In this study, eCO_2_ resulted in increased phytic acid concentrations (PAC) by 2.4% on average, which is in agreement with previous studies (Myers et al., 2014; Zhou et al., 2014; Niu et al., 2021). Although in most studies, this increase was not statistically significant, this trend potentially affects the nutritional value of rice by reducing the bioavailability of microelements such as Zn and Fe for the consumer. In this study, the PAC of brown rice was, on average, more than 7 times that of milled rice (Figure 5), mainly because the accumulation of phytic acid in aleurone accounts for more than 85% of that in the whole grain (Wang et al., 2009). In this sense, the phytic acid concentration of brown rice might be more noteworthy. Compared with CK, LC significantly increased the PAC by 4.3% on average, mainly because of a large increase in brown rice and a slight decrease in milled rice. During phytic acid synthesis, inositol and inorganic phosphorus are first synthesized to inositol monophosphate by phytic acid synthetase and then to phytic acid after a series of reactions; therefore, most of the P in grains is stored in the cortex and embryo in the form of phytic acid (Phillippy, 1998). In the present study, LC treatment significantly increased the P concentration of brown rice by 8.4% but did not affect that of milled rice, which may be one of the reasons for the significant increase of PAC in brown rice compared with milled rice.

We observed a significant year effect on rice quality indicators. For example, in 2017, the MRP and HRP values were lower, whereas the chalkiness was significantly higher than in the other two seasons. Although the average temperatures of the whole three growing seasons were close (Supplementary Figure 1), we observed that the average daily temperature during the early grain-filling stage (1 week after heading) in 2017 reached 28.3°C, which was 0.9°C and 1.2°C higher than that in 2016 and 2018, respectively. This may have caused an accelerated grain-filling rate during the early grain-filling stage, which can induce grain chalkiness (Yang et al., 2007; Usui et al., 2014; Zhang et al., 2015). Overall, cooking and eating quality in 2018 was higher compared to 2016 or 2017, which may be related to sufficient monthly average sunshine duration (about 200 h) in the growth period and low rainfall in the late grain-filling stage (September to October). We observed a significant interaction between LC and year for rice quality, in particular, LC largely affected chalkiness and RVA parameters in 2018, whereas the effects of LC on eating quality parameters were smaller, indicating that the effects of source reduction treatment (LC) on rice quality are related to climatic conditions. On the contrary, there were far few interactions between CO_2_ and year in this study, indicating CO_2_ effects on rice quality less affected by different years.

## Conclusion

In this study, we artificially reduced the source-sink ratio of rice *via* cutting off leaves, and the effects of eCO_2_ and LC treatment on rice quality were observed under field conditions. Our results show that the processing and appearance qualities of rice were significantly decreased by elevated atmospheric CO_2_ concentrations, and the nutritional quality (especially regarding the levels of protein, Ca, S, and Cu) was also decreased, whereas the eating and cooking quality was improved. Compared with the CK treatment, LC treatment itself had no significant effects on processing or eating quality but significantly increased the chalkiness and the levels of protein and minerals, albeit to different degrees. Grain chalkiness increased under both eCO_2_ (increased source) and LC (reduced source), which despite appearing to be a contradiction might be explained by thermal treatment effects of eCO_2_ also causing source limitation at the grain filling stage, but this requires further research. The effect of the LC treatment on eating quality was significantly lower than that of eCO_2_. Overall, reducing the source-sink ratio of rice *via* LC treatment cannot significantly change the response of rice quality traits to eCO_2_.

## Data Availability Statement

The original contributions presented in the study are included in the article/Supplementary Material, further inquiries can be directed to the corresponding author/s.

## Author Contributions

BG: conceptualization, methodology, data curation, investigation, and writing – original draft. SH, KW, HL, and XS: data curation. LJ: data curation and funding acquisition. YXW: supervision, validation, and funding acquisition. JZ: supervision and validation. YLW: supervision, writing – review and editing, supervision, and funding acquisition. LY: conceptualization, methodology, writing – review and editing, supervision, and funding acquisition. All authors contributed to the article and approved the submitted version.

## Conflict of Interest

The authors declare that the research was conducted in the absence of any commercial or financial relationships that could be construed as a potential conflict of interest.

## Publisher’s Note

All claims expressed in this article are solely those of the authors and do not necessarily represent those of their affiliated organizations, or those of the publisher, the editors and the reviewers. Any product that may be evaluated in this article, or claim that may be made by its manufacturer, is not guaranteed or endorsed by the publisher.
