# Supplementary material for: Impact of Elevated CO2 and Reducing the Source-Sink Ratio by Partial Defoliation on Rice Grain Quality – A 3-Year Free-Air CO2 Enrichment Study
Source: Front Plant Sci. 2021 Dec 23;12:788104. doi: 10.3389/fpls.2021.788104 (PMC8733338; doi:10.3389/fpls.2021.788104)
Supplement: Supplementary file 1 [file Table_1.DOCX]

Impact of elevated CO_2_ and reducing the source-sink ratio by partial defoliation on rice grain quality – A 3-year free-air CO_2_ enrichment study

Bo Gao^a,b^, Shaowu Hu^a^, Liquan Jing^a^, Yunxia Wang^c^, Jianguo Zhu^d^, Kai Wang^b^, Hongyang Li^b^, Xingxing Sun^b^, Yulong Wang^a,*^, Lianxin Yang^a,*^

^a^ *Key Laboratory of Crop Genetics and Physiology of Jiangsu Province/Co-Innovation Center for Modern Production Technology of Grain Crops of Jiangsu Province, Yangzhou University, Yangzhou 225009, Jiangsu, China*

^b^ *Jiangsu Coastal Area Institute of Agricultural Sciences, Yancheng 224002, Jiangsu, China*

^c^ *College of Environmental Science and Engineering, Yangzhou University, Yangzhou 225009, Jiangsu, China*

^d^ *State Key Laboratory of Soil and Sustainable Agriculture, Institute of Soil Science, Chinese Academy of Sciences, Nanjing 210008, Jiangsu, China*

* Corresponding authors: Email: ylwang@yzu.edu.cn; lxyang@yzu.edu.cn.

**Supplementary materials**

**Fig. S1.** Daily maximum, minimum and mean temperatures from transplanting to maturity during 2016 (a)、2017 (b) and 2018 (c) rice growing seasons. Arrows ① indicated transplanting dates; arrows ② and ③ indicated flowering dates for FACE and ambient plots, respectively; arrows ④ and ⑤ indicated maturity dates for FACE and ambient plots, respectively.

**Fig. S2.** Mean monthly sunshine duration and precipitation during 2016-2018 rice growing seasons.

**Table S1**

Significance of elevated CO_2_, year, cutting off top three leaves (LC), and their interactions on various of rice chalky grain percentage and chalkiness degree from the 2016-2018 experiments.

| Parameters | Chalky grain percentage (%) | Chalkiness degree (%) |
| --- | --- | --- |
| Year | ** | ** |
| CO_2_ | **↑ | **↑ |
| LC | **↑ | **↑ |
| CO_2_ × Year | ns | ns |
| CO_2_ × LC | ns | ns |
| LC × Year | * | ** |
| CO_2_ × LC × Year | ns | ns |

Arrows in the treatment column indicate treatment increased (↑) or decreased (↓) the values. Statistically significant effects are indicated as ** *P* < 0.01; * *P* < 0.05; ns, not significant.

**Table S2**

Effect of elevated CO_2_ and cutting off top three leaves (LC) on concentrations of macro and micro elements in **milled rice** over three cropping seasons (2016–2018). AC and EC refer to ambient CO_2_ and elevated CO_2_, respectively.

| Year | Treatment | CO_2_ | Ca  (mg g^-1^) | K  (mg g^-1^) | Mg  (mg g^-1^) | P  (mg g^-1^) | S  (mg g^-1^) | B  (mg kg^-1^) | Cu  (mg kg^-1^) | Fe  (mg kg^-1^) | Mn  (mg kg^-1^) | Zn  (mg kg^-1^) |
| --- | --- | --- | --- | --- | --- | --- | --- | --- | --- | --- | --- | --- |
| 2016 | CK | AC | 0.13±0.00 | 0.71±0.02 | 0.15±0.00 | 0.75±0.01 | 0.86±0.01 | 1.04±0.09 | 3.41±0.27 | 23.98±1.60 | 11.83±0.29 | 15.31±0.87 |
|  |  | EC | 0.10±0.01 | 0.72±0.05 | 0.16±0.01 | 0.75±0.03 | 0.81±0.01 | 1.10±0.10 | 3.16±0.13 | 22.57±2.28 | 10.74±0.96 | 14.17±0.17 |
|  |  | % change | -26.0 | 1.8 | 9.4 | 0.0 | -5.2 | 5.2 | -7.4 | -5.9 | -9.2 | -7.5 |
|  | LC | AC | 0.13±0.01 | 0.70±0.03 | 0.14±0.00 | 0.77±0.01 | 0.95±0.02 | 1.32±0.03 | 3.46±0.39 | 27.75±1.71 | 12.55±0.99 | 17.53±1.21 |
|  |  | EC | 0.12±0.00 | 0.74±0.01 | 0.15±0.01 | 0.78±0.02 | 0.89±0.01 | 1.42±0.14 | 3.64±0.19 | 28.03±0.93 | 13.68±0.36 | 18.87±1.64 |
|  |  | % change | -7.6 | 6.4 | 5.4 | 0.8 | -6.1 | 7.7 | 5.4 | 1.0 | 9.0 | 7.6 |
| 2017 | CK | AC | 0.14±0.01 | 0.77±0.02 | 0.27±0.02 | 0.98±0.02 | 0.98±0.01 | 1.07±0.07 | 2.87±0.23 | 22.05±2.50 | 15.74±1.60 | 16.41±0.58 |
|  |  | EC | 0.13±0.00 | 0.88±0.04 | 0.30±0.01 | 1.01±0.03 | 1.01±0.01 | 1.19±0.12 | 2.38±0.27 | 26.14±2.48 | 13.40±0.47 | 15.88±0.67 |
|  |  | % change | -3.3 | 13.5 | 10.5 | 2.9 | 2.4 | 10.6 | -17.1 | 18.5 | -14.9 | -3.3 |
|  | LC | AC | 0.19±0.00 | 0.76±0.04 | 0.30±0.02 | 1.06±0.04 | 1.08±0.02 | 1.66±0.16 | 3.09±0.03 | 31.52±3.65 | 15.77±0.93 | 21.39±0.37 |
|  |  | EC | 0.17±0.00 | 0.84±0.03 | 0.31±0.02 | 1.09±0.03 | 1.06±0.02 | 1.46±0.03 | 3.06±0.33 | 31.53±2.89 | 13.63±0.62 | 21.77±0.11 |
|  |  | % change | -11.6 | 9.3 | 3.9 | 2.8 | -2.4 | -12.0 | -1.0 | 0.0 | -13.5 | 1.8 |
| 2018 | CK | AC | 0.14±0.01 | 1.04±0.09 | 0.44±0.03 | 1.33±0.07 | 1.04±0.01 | 3.36±0.27 | 4.20±0.48 | 18.70±1.35 | 13.27±0.77 | 17.07±0.94 |
|  |  | EC | 0.12±0.00 | 1.05±0.02 | 0.46±0.03 | 1.39±0.05 | 1.04±0.02 | 2.97±0.07 | 4.14±0.34 | 16.21±1.57 | 14.77±1.23 | 17.15±1.67 |
|  |  | % change | -9.9 | 1.2 | 5.7 | 4.6 | -0.8 | -11.7 | -1.5 | -13.3 | 11.3 | 0.5 |
|  | LC | AC | 0.12±0.01 | 1.04±0.00 | 0.42±0.03 | 1.38±0.06 | 1.16±0.04 | 2.79±0.11 | 4.74±0.21 | 17.39±2.50 | 12.91±0.44 | 19.03±0.08 |
|  |  | EC | 0.11±0.01 | 0.94±0.05 | 0.37±0.02 | 1.28±0.03 | 1.06±0.03 | 2.70±0.07 | 4.46±0.09 | 17.68±1.19 | 12.61±0.35 | 19.91±0.56 |
|  |  | % change | -4.3 | -10.2 | -11.7 | -7.1 | -8.6 | -3.1 | -5.9 | 1.7 | -2.4 | 4.6 |
| ANOVA |  |  |  |  |  |  |  |  |  |  |  |  |
| Year |  |  | ** | ** | ** | ** | ** | ** | ** | ** | ** | ** |
| CO_2_ |  |  | **↓ | ns | ns | ns | **↓ | ns | ns | ns | ns | ns |
| LC |  |  | **↑ | ns | ns | ns | **↑ | ns | *↑ | **↑ | ns | **↑ |
| CO_2_ × Year |  |  | ns | 0.071 | ns | ns | ns | ns | ns | ns | 0.061 | ns |
| CO_2_ × LC |  |  | ns | ns | ns | ns | * | ns | ns | ns | ns | ns |
| LC × Year |  |  | ** | ns | * | ns | ns | ** | ns | 0.077 | 0.052 | 0.072 |
| CO_2_ × LC × Year |  |  | 0.071 | ns | ns | ns | ns | ns | ns | ns | ns | ns |

Arrows in the treatment column indicate treatment increased (↑) or decreased (↓) the values. Values are means ± standard error (n = 3); Statistically significant effects are indicated as ** *P* < 0.01; * *P* < 0.05. The value indicates the probability between 0.05 and 0.1. ns, not significant.

**Table S3**

Effect of elevated CO_2_ and cutting off top three leaves (LC) on concentrations of macro and micro elements in **brown rice** over three cropping seasons (2016–2018). AC and EC refer to ambient CO_2_ and elevated CO_2_, respectively.

| Year | Treatment | CO_2_ | Ca  (mg g^-1^) | K  (mg g^-1^) | Mg  (mg g^-1^) | P  (mg g^-1^) | S  (mg g^-1^) | B  (mg kg^-1^) | Cu  (mg kg^-1^) | Fe  (mg kg^-1^) | Mn  (mg kg^-1^) | Zn  (mg kg^-1^) |
| --- | --- | --- | --- | --- | --- | --- | --- | --- | --- | --- | --- | --- |
| 2016 | CK | AC | 0.17±0.00 | 2.80±0.04 | 1.31±0.04 | 3.81±0.03 | 1.20±0.02 | 1.58±0.08 | 6.42±0.52 | 41.21±1.54 | 42.94±1.15 | 23.81±0.75 |
|  |  | EC | 0.17±0.01 | 2.86±0.07 | 1.36±0.06 | 3.91±0.09 | 1.19±0.02 | 1.52±0.14 | 6.39±0.31 | 45.73±5.37 | 41.04±3.62 | 25.81±0.51 |
|  |  | % change | -3.76 | 2.32 | 3.80 | 2.53 | -0.26 | -3.86 | -0.58 | 10.99 | -4.42 | 8.40 |
|  | LC | AC | 0.17±0.01 | 2.84±0.03 | 1.41±0.01 | 4.10±0.01 | 1.31±0.06 | 2.12±0.32 | 7.44±0.28 | 39.25±4.05 | 42.62±3.28 | 28.22±1.22 |
|  |  | EC | 0.19±0.01 | 2.91±0.11 | 1.42±0.02 | 4.21±0.07 | 1.28±0.04 | 1.52±0.14 | 6.47±0.31 | 48.58±5.20 | 46.53±1.56 | 27.87±0.29 |
|  |  | % change | 11.71 | 2.32 | 0.65 | 2.54 | -2.27 | -28.30 | -13.02 | 23.78 | 9.17 | -1.21 |
| 2017 | CK | AC | 0.21±0.01 | 2.87±0.03 | 1.43±0.02 | 4.01±0.03 | 1.24±0.01 | 1.60±0.07 | 5.14±0.46 | 36.78±1.84 | 46.09±2.85 | 30.39±1.92 |
|  |  | EC | 0.21±0.01 | 3.02±0.02 | 1.41±0.06 | 3.99±0.09 | 1.27±0.03 | 1.83±0.18 | 4.11±0.54 | 35.37±0.70 | 44.07±0.97 | 29.23±1.72 |
|  |  | % change | 1.71 | 5.01 | -1.25 | -0.41 | 2.02 | 14.91 | -19.97 | -3.82 | -4.39 | -3.82 |
|  | LC | AC | 0.23±0.02 | 3.17±0.07 | 1.47±0.01 | 4.20±0.06 | 1.34±0.05 | 1.83±0.21 | 5.14±0.73 | 40.82±4.39 | 46.90±5.23 | 34.27±1.14 |
|  |  | EC | 0.22±0.00 | 3.42±0.08 | 1.47±0.03 | 4.25±0.10 | 1.30±0.03 | 1.65±0.02 | 4.17±0.63 | 39.17±2.77 | 45.69±1.66 | 31.24±1.32 |
|  |  | % change | -5.15 | 7.83 | 0.31 | 1.22 | -2.78 | -9.83 | -18.95 | -4.03 | -2.58 | -8.86 |
| 2018 | CK | AC | 0.19±0.00 | 3.46±0.05 | 1.83±0.02 | 4.60±0.05 | 1.20±0.02 | 5.78±0.05 | 5.12±0.61 | 29.10±2.41 | 34.48±1.30 | 23.57±0.98 |
|  |  | EC | 0.20±0.01 | 3.53±0.08 | 1.88±0.04 | 4.72±0.07 | 1.19±0.01 | 5.32±0.08 | 4.64±0.40 | 27.20±1.31 | 38.91±2.96 | 25.15±1.70 |
|  |  | % change | 5.52 | 2.21 | 2.60 | 2.44 | -0.79 | -2.89 | -9.39 | -6.53 | 12.83 | 6.70 |
|  | LC | AC | 0.19±0.01 | 3.66±0.06 | 1.92±0.04 | 5.13±0.11 | 1.33±0.04 | 5.21±0.17 | 5.34±0.33 | 28.93±3.75 | 36.13±0.31 | 26.92±0.32 |
|  |  | EC | 0.19±0.00 | 3.74±0.05 | 1.92±0.01 | 5.20±0.04 | 1.29±0.02 | 5.46±0.16 | 5.08±0.37 | 30.72±2.73 | 37.24±2.15 | 28.60±0.95 |
|  |  | % change | -4.05 | 2.07 | 0.24 | 1.28 | -3.03 | 4.80 | -4.96 | 6.17 | 3.07 | 6.27 |
| ANOVA |  |  |  |  |  |  |  |  |  |  |  |  |
| Year |  |  | ** | ** | ** | ** | ns | ** | ** | ** | ** | ** |
| CO_2_ |  |  | ns | **↑ | ns | ns | ns | ns | *↓ | ns | ns | ns |
| LC |  |  | ns | **↑ | **↑ | **↑ | **↑ | ns | ns | ns | ns | **↑ |
| CO_2_ × Year |  |  | ns | ns | ns | ns | ns | ns | ns | ns | ns | 0.085 |
| CO_2_ × LC |  |  | ns | ns | ns | ns | ns | ns | ns | ns | ns | ns |
| LC × Year |  |  | 0.063 | ** | ns | * | ns | ns | ns | ns | ns | ns |
| CO_2_ × LC × Year |  |  | ns | ns | ns | ns | ns | 0.086 | ns | ns | ns | ns |

Arrows in the treatment column indicate treatment increased (↑) or decreased (↓) the values. Values are means ± standard error (n = 3); Statistically significant effects are indicated as ** *P* < 0.01; * *P* < 0.05. The value indicates the probability between 0.05 and 0.1. ns, not significant.

**Table S4**

Significance of year, positions, elevated CO_2_, cutting off top three leaves (LC), and their interactions on various of rice nutritional quality from the 2016-2018 experiments.

| Parameters | Protein(mg·g^-1^) | Ca(mg/g) | K(mg/g) | Mg(mg/g) | P(mg/g) | S(mg/g) | B(mg/kg) | Cu(mg/kg) | Fe(mg/kg) | Mn(mg/kg) | Zn(mg/kg) | Phytic acid (mg·g^-1^) |
| --- | --- | --- | --- | --- | --- | --- | --- | --- | --- | --- | --- | --- |
| Year (Y) | ** | ** | ** | ** | ** | ** | ** | ** | ** | ** | ** | ** |
| CO_2_ | **↓ | *↓ | **↑ | ns | ns | *↓ | ns | *↓ | ns | ns | ns | ns |
| LC | **↑ | **↑ | **↑ | *↑ | **↑ | **↑ | ns | *↑ | *↑ | ns | **↑ | **↑ |
| Position (P) | **↑ | **↑ | **↑ | **↑ | **↑ | **↑ | **↑ | **↑ | **↑ | **↑ | **↑ | **↑ |
| CO_2_×Y | ns | ns | * | ns | ns | ns | ns | ns | ns | ns | ns | * |
| CO_2_×LC | ns | ns | ns | ns | ns | 0.056 | ns | ns | ns | ns | ns | ns |
| CO_2_×P | ns | * | * | ns | ns | ns | ns | ns | ns | ns | ns | ns |
| LC×Y | ns | ** | * | ns | ns | ns | ** | ns | ns | ns | ns | ns |
| LC×P | ns | ns | ** | ** | ** | ns | ns | ns | ns | ns | ns | ** |
| P×Y | ns | ns | ** | ** | ** | ** | ** | ** | * | ** | ** | ** |
| CO_2_×L×Y | ns | * | ns | ns | ns | ns | * | ns | ns | ns | ns | 0.054 |
| CO_2_×P×Y | ns | ns | ns | ns | ns | ns | 0.051 | ns | ns | ns | ns | 0.054 |
| CO_2_×LC×P | ns | ns | ns | ns | ns | ns | ns | ns | ns | ns | ns | ns |
| LC×P×Y | ns | 0.068 | ** | ns | ** | ns | * | ns | ns | ns | ns | ns |
| CO_2_×LC×P×Y | ns | ns | ns | ns | ns | ns | ns | ns | ns | ns | ns | ns |

Arrows in the treatment of C and LC columns indicate treatment increased (↑) or decreased (↓) the values. Arrows (↑) in the treatment of P column indicate the values in brown rice are greater than those in milled rice. Statistically significant effects are indicated as ** *P* < 0.01; * *P* < 0.05. The value indicates the probability between 0.05 and 0.1. ns, not significant.

**Table S5**

Correlation matrix of protein concentration, amylose concentration, traits of RVA and taste of cooked milled rice under elevated CO_2_ and cutting off top three leaves over three cropping seasons (2016–2018).

| Parameters | PC | AC | PV | MV | BD | FV | SB | CS | PT | GT | Appearance | Hardness | Stickiness | Balance degree | OPI |
| --- | --- | --- | --- | --- | --- | --- | --- | --- | --- | --- | --- | --- | --- | --- | --- |
| PC | 1 |  |  |  |  |  |  |  |  |  |  |  |  |  |  |
| AC | 0.004 | 1 |  |  |  |  |  |  |  |  |  |  |  |  |  |
| PV | 0.143 | 0.689^*^ | 1 |  |  |  |  |  |  |  |  |  |  |  |  |
| MV | 0.333 | 0.550^+^ | **0.951**^**^ | 1 |  |  |  |  |  |  |  |  |  |  |  |
| BD | -0.424 | **0.688**^*^ | **0.60**2^*^ | 0.327 | 1 |  |  |  |  |  |  |  |  |  |  |
| FV | 0.167 | **0.907**^**^ | **0.912**^**^ | **0.838**^**^ | **0.628**^*^ | 1 |  |  |  |  |  |  |  |  |  |
| SB | 0.077 | **0.618**^*^ | -0.084 | -0.154 | 0.139 | 0.331 | 1 |  |  |  |  |  |  |  |  |
| CS | -0.181 | **0.852**^**^ | 0.276 | 0.069 | **0.668**^*^ | **0.603**^*^ | **0.830**^**^ | 1 |  |  |  |  |  |  |  |
| PT | -0.018 | -0.002 | 0.515^+^ | **0.581**^*^ | 0.075 | 0.303 | -0.449 | -0.295 | 1 |  |  |  |  |  |  |
| GT | -0.193 | 0.052 | -0.279 | -0.229 | -0.264 | -0.159 | 0.256 | 0.044 | -0.414 | 1 |  |  |  |  |  |
| Appearance | -0.457 | -0.062 | 0.288 | 0.200 | 0.365 | 0.029 | **-0.592**^*^ | -0.239 | 0.449 | -0.119 | 1 |  |  |  |  |
| Hardness | **0.579**^*^ | 0.110 | -0.239 | -0.171 | -0.290 | 0.024 | **0.607**^*^ | 0.293 | -0.414 | 0.043 | **-0.904**^**^ | 1 |  |  |  |
| Stickiness | **-0.793**^**^ | -0.062 | 0.110 | -0.040 | 0.440 | -0.082 | -0.452 | -0.092 | 0.240 | 0.112 | **0.877**^**^ | **-0.874**^**^ | 1 |  |  |
| Balance degree | **-0.695**^*^ | -0.481 | -0.307 | -0.373 | 0.026 | -0.522^+^ | -0.562^+^ | -0.408 | 0.163 | 0.114 | **0.764**^**^ | **-0.786**^**^ | **0.839**^**^ | 1 |  |
| OPI | **-0.626**^*^ | **-0.673**^*^ | -0.576^+^ | **-0.606**^*^ | -0.196 | **-0.757**^**^ | -0.515^+^ | -0.498 | -0.082 | 0.244 | 0.508^+^ | **-0.593**^*^ | **0.670**^*^ | **0.927**^**^ | 1 |

PC, protein concentration; AC, amylose concentration; PV, peak viscosity; MV, minimum viscosity; BD, breakdown; FV, final viscosity; SB, setback, CS, consistence; PT, peak time; GT, gelatinization temperature; OPI, Overall palatability index. ^**^ *P* < 0.01, ^*^ *P* < 0.05, ^+^ *P* < 0.1, n = 12.
